# Impacts of Alternative Billing Claims on Hypertension Prevalence and Mortality Estimates in Alberta, Canada

**DOI:** 10.23889/ijpds.v1i1.379

**Published:** 2017-04-19

**Authors:** Ceara Tess Cunningham, Hude Quan, Nathalie Jette, Guanmin Chen

**Affiliations:** 1 Alberta Health Services; 2 University of Calgary

## Objectives

In Canada, secondary sources of health information such as physician billing claims are used for surveillance of chronic conditions such as hypertension. The value of this data may be affected by different types of physician payment models. Fee-for-service (FFS) physicians submit billing claims in order to be remunerated whereas physicians on salary or capitation also called alternative payment plans (APP) are remunerated regardless of whether claims are submitted. Thus there are concerns nationally that APPs may be associated with decreased frequency of billing submission, thereby eroding data quality and leading to underestimates of disease burdens and outcomes. We assessed the impact of APPs on hypertension prevalence, mortality and cardiovascular (CVD) hospitalization in Alberta.

## Approach

The following health administrative databases from Alberta, Canada were used in this study: 1) Alberta Health Care Insurance Plan registry; 2) Hospital Discharge Abstract database (DAD); 3) Physician claims data and; 4) Vital Statistics database. We defined patients with hypertension aged 20 years and older between April 1, 2004 and March 31, 2009 (fiscal years 2004 to 2009) based on previously validated algorithm. Cases were then sorted into FFS and non-FFS billing status. Descriptive statistics for age group, sex, income quintile, rural/urban geographical location, death status and comorbidities were reported between the shadow billing and non-shadow billing groups in regards to prevalence, all-cause mortality and CVD hospitalizations.

## Results

 A total of 613, 844 adult hypertensive cases were captured during the 5 year study period. The majority of hypertension cases (99.6%, n=610,167) were identified through FFS claims. The effect of non-FFS billing estimates represented a small proportion of hypertension cases (0.40%, n=3677) identified during the study period. Among the FFS claims, overall hypertension prevalence was 22.2% (n=610167) and the effect of non-FFS billing estimates (0.13%, n=3677) on prevalence estimates was relatively minor. All-cause mortality 33.8 (95% CI 33.6-34) and CVD 40.6 (40.4-40.9) were higher for FFS cases than non-FFS cases 19.0 (16.6-21.8) and CVD: 8.0 (6.4-9.8).

## Conclusion

During the study time period, the impact of non-FFS (i.e. shadow billings) on physician claims may not have impacted hypertension prevalence estimates greatly. Since Alberta uses shadow billing incentive programs, future research is needed to determine whether incentive programs should be considered in other provinces or nationally in order to preserve the overall quality of physician claims data.

